# Zinc rescues obesity‐induced cardiac hypertrophy *via* stimulating metallothionein to suppress oxidative stress‐activated BCL10/CARD9/p38 MAPK pathway

**DOI:** 10.1111/jcmm.13050

**Published:** 2017-02-03

**Authors:** Shudong Wang, Junlian Gu, Zheng Xu, Zhiguo Zhang, Tao Bai, Jianxiang Xu, Jun Cai, Gregory Barnes, Qiu‐Ju Liu, Jonathan H. Freedman, Yonggang Wang, Quan Liu, Yang Zheng, Lu Cai

**Affiliations:** ^1^ Cardiovascular CenterThe First Hospital of Jilin UniversityChangchunJilinChina; ^2^ Department of PediatricsKosair Children's Hospital Research InstituteUniversity of LouisvilleLouisvilleKYUSA; ^3^ Autism CenterUniversity of LouisvilleLouisvilleKYUSA; ^4^ Department of Hematology DisordersThe First Hospital of Jilin UniversityChangchunJilinChina; ^5^ Department of Pharmacology and ToxicologyUniversity of LouisvilleLouisvilleKYUSA; ^6^ Wendy Novak Diabetes Care CenterUniversity of LouisvilleLouisvilleKYUSA

**Keywords:** obesity‐related cardiac hypertrophy, zinc, BCL10, CARD9, p38 MAPK

## Abstract

Obesity often leads to obesity‐related cardiac hypertrophy (ORCH), which is suppressed by zinc‐induced inactivation of p38 mitogen‐activated protein kinase (p38 MAPK). In this study, we investigated the mechanisms by which zinc inactivates p38 MAPK to prevent ORCH.

Mice (4‐week old) were fed either high fat diet (HFD, 60% kcal fat) or normal diet (ND, 10% kcal fat) containing variable amounts of zinc (deficiency, normal and supplement) for 3 and 6 months. P38 MAPK siRNA and the p38 MAPK inhibitor SB203580 were used to suppress p38 MAPK activity *in vitro and in vivo*, respectively. HFD activated p38 MAPK and increased expression of B‐cell lymphoma/CLL 10 (BCL10) and caspase recruitment domain family member 9 (CARD9). These responses were enhanced by zinc deficiency and attenuated by zinc supplement. Administration of SB203580 to HFD mice or specific siRNA in palmitate‐treated cardiomyocytes eliminated the HFD and zinc deficiency activation of p38 MAPK, but did not significantly impact the expression of BCL10 and CARD9. In cultured cardiomyocytes, inhibition of BCL10 expression by siRNA prevented palmitate‐induced increased p38 MAPK activation and atrial natriuretic peptide (ANP) expression. In contrast, inhibition of p38 MAPK prevented ANP expression, but did not affect BCL10 expression. Deletion of metallothionein abolished the protective effect of zinc on palmitate‐induced up‐regulation of BCL10 and phospho‐p38 MAPK. HFD and zinc deficiency synergistically induce ORCH by increasing oxidative stress‐mediated activation of BCL10/CARD9/p38 MAPK signalling. Zinc supplement ameliorates ORCH through activation of metallothionein to repress oxidative stress‐activated BCL10 expression and p38 MAPK activation.

## Introduction

Obesity has become a global epidemic affecting both paediatric and adult populations. Previous research has been mainly focused on the effects of obesity on adult health, morbidity and mortality 1. However, obese children have a high risk of developing obesity at adulthood, in addition to other health issues including diabetes and cardiovascular disease. Early intervention can potentially reduce these risks. Therefore, it is critical to find effective therapies to prevent childhood obesity‐induced health outcome 2. p38 mitogen‐activated protein kinase (p38 MAPK) is one of the serine/threonine kinase families that is activated in response to various extracellular stimuli to modulate the function of multiple transcription activators 3, 4. p38 MAPK activation contributes to cardiac hypertrophy and dysfunction under various conditions 4, 5 which indicates a significant role for p38 MAPK in the initiation and development of cardiac hypertrophy. In addition, our previous results have shown that inactivation of p38 MAPK prevents the development of cardiac hypertrophy in obese mice 6.

Caspase recruitment domain family member 9 (CARD9), an adaptor molecule harbouring an N‐terminal caspase‐binding domain (CARD) and a C‐terminal coiled‐coil domain 7, is involved in inflammation and immune activation 8. Indeed, overexpression of CARD9 strongly activates p38 MAPK, which is pivotal in a multitude of immune responses 9. B‐cell lymphoma/CLL 10 (BCL10) was first identified as a target of a chromosomal translocation in mucosa‐associated lymphoid tissue lymphoma 10 and was linked to normal lymphocyte function 11. BCL10 has also been shown to mediate inflammatory responses in several cell types including hepatocytes and mouse embryonic fibroblasts 12, 13. Bcl10 expression is linked to carrageenan‐induced chronic inflammation and insulin resistance 14, 15. The BCL10–CARD9 complex is also required for mediating inflammation 7, 16, suggesting a functional interaction between BCL10 and CARD9.

Zinc is structurally and functionally required for more than 2000 transcription factors. Recently, studies have revealed that zinc supplementation provides some benefit to diabetic and non‐diabetic obese patients, with similar effects in animal models 17, 18. We previously demonstrated that zinc attenuated the destructive effect of diabetes on heart, kidney, liver and testis by enhancing insulin sensitivity and reducing oxidative stress and inflammation 19, 20. As a powerful antioxidant, metallothionein (MT) ameliorates diabetic cardiomyopathy and nephropathy 21, 22. Metallothionein expression is up‐regulated by zinc and contributes to the protection against diabetes‐induced cardiomyopathy 22, 23. These data suggest that MT is involved in the mechanisms underpinning zinc‐associated cardiac protection. We demonstrated that zinc supplementation plays an important role in attenuating ORCH 6. In the same study, we also presented evidence that ORCH is exacerbated by zinc deficiency and that this response may be mediated by up‐regulation of the p38 MAPK‐mediated inflammation pathway. However, the mechanism by which zinc regulates p38 activity remains unclear. In this study, we elucidated the molecular mechanism underlying zinc‐mediated inactivation of p38 MAPK pathway in both the ORCH animal model and cultured cardiomyocytes. We hypothesize that (1) obesity activates p38 MAPK pathway through the upregulation of Bcl10/CARD9 complex. Additionally, zinc supplementation will inhibit Bcl10/CARD9‐mediated p38 MAPK activation whereas zinc deficiency will further activate the pathway; (2) obesity‐induced expression of Bcl10/CARD9 is mediated by increased oxidative stress, which can be enhanced by zinc deficiency or ameliorated by zinc supplement; and (3) the prevention of obesity‐induced oxidative stress and subsequently up‐regulation of Bcl10/CARD9 expression by zinc supplement is mediated by up‐regulation of MT as a potent antioxidant. To test this hypothesis, we have used the same animal model that was used in a previous study, in which p38 MAPK inhibitor was used to perturb p38 pathway 6. In addition, primary cultures of adult cardiomyocytes with p38α MAPK‐ and Bcl10‐specific siRNA were used for mechanistic studies.

## Materials and methods

### Animals

C57BL/6J male mice (3‐week old, immediately after weaning) were obtained from the Jackson Laboratory (Bar Harbor, Maine). Mice were raised at the University of Louisville Research Resources Center at 22°C with a 12:12 hr light:dark cycle and consumed tap water and standard rodent diet unless otherwise stated. All experimental procedures were in compliance with the Guide for the Care and Use of Laboratory Animals, Eighth Edition (Library of Congress Control Number: 2010940400, revised 2011) and approved by the Institutional Animal Care and Use Committee of the University of Louisville (No. 12112).

### Establishment of obese mouse model and treatment with zinc

Establishment of the obese mouse model and treatment with different amounts of zinc were described previously 6. Briefly, one week after arriving, mice were fed either HFD (60% kcal fat, Research Diets, New Brunswick, NJ, USA) or (ND, 10% kcal fat) containing one of three different quantities of zinc (zinc deficiency, ZD; normal zinc, ZN; or zinc supplement, ZS) for 3 or 6 months, respectively. Mice were randomly assigned into one of six groups as follows: ND/ZN (*n* = 10), ND/ZD (*n* = 10), ND/ZS (*n* = 12), HFD/ZN (*n* = 14), HFD/ZD (*n* = 16) and HFD/ZS (*n* = 16). Mice were killed under anaesthesia with an intraperitoneal injection of avertin (250 mg/kg) after 3 or 6 months of feeding, corresponding to juvenile (4 months old) and young adult (7 months old), respectively 24.

A mechanistic study was performed with five groups of mice: ND/ZN (or Ctrl, *n* = 6), HFD/ZN (*n* = 6), HFD/ZN/SB treated with p38 MAPK inhibitor for 3 months (SB203580, 1 mg/kg bodyweight, intraperitoneal injection, at the beginning of HFD treatment, *n* = 7), HFD/ZD (*n* = 7) and HFD/ZD/SB treated with SB203580 (*n* = 8).

### Western blotting

Western blotting was performed as previously described 25, 26 (supporting information). Metallothionein expression was determined using a modified Western blot procedure, as described previously 22.

### Quantitative real‐time PCR

qRT‐PCR was performed following our published protocols 22, 27 (supporting information).

### Co‐immunoprecipitation of BCL10–CARD9 complex

Co‐immunoprecipitation was performed as described previously 25 (supporting information).

### Immunofluorescent staining

Immunofluorescent staining was performed, as previously described 28. Images were captured with Nikon microscope (Nikon, Melville, NY, USA). Positively staining nuclei were scored in experimental groups and compared to that in the control group (ND/NZ‐treated mice).

### Primary cardiomyocyte isolation, culture, treatment with palmitate, zinc and TPEN and small interfering RNA transfection

Primary adult mouse cardiomyocytes were isolated from 2‐month‐old male MT1/2‐KO (MT‐KO) and 129S1 mice (the Jackson Laboratory, Bar Harbor, Maine) and cultured as previously described 29. Primary cardiomyocytes were treated with palmitate at 100 μM to mimic *in vivo* HFD and with either 2 μM TPEN, a zinc chelator or 50 μM zinc chloride (Sigma‐Aldrich, St. Louis, MO, USA) to mimic zinc deficiency and zinc supplement, respectively. Primary cardiomyocytes were also pre‐treated with specific small interfering RNA (siRNA) for either p38 MAPK or BCL10 to down‐regulate their expression. Specific siRNA targeting against p38 MAPK (MSS240941) and BCL10 (MSS202346) was purchased from Invitrogen (Waltham, MA, USA). Negative control siRNAs with no sequence homology to any known mouse genes were used as a control.

### Statistical analysis

Data, collected from three replicates of cell‐culture experiments or from 5 to 8 animals in each experimental group, are presented as mean ± standard deviation. One‐way or two‐way anova were used to evaluate statistically significant differences among multiple groups, followed by post hoc pairwise repetitive comparisons with Tukey test to evaluate the significant difference between two groups (Origin 8.0 Lab data analysis and graphing software). Statistical significance was considered as *P* < 0.05.

## Results

### Obesity‐induced expression of p38 MAPK, BCL10 and CARD9 in the heart was exacerbated and prevented by zinc deficiency and supplement, respectively

**Figure 1:**
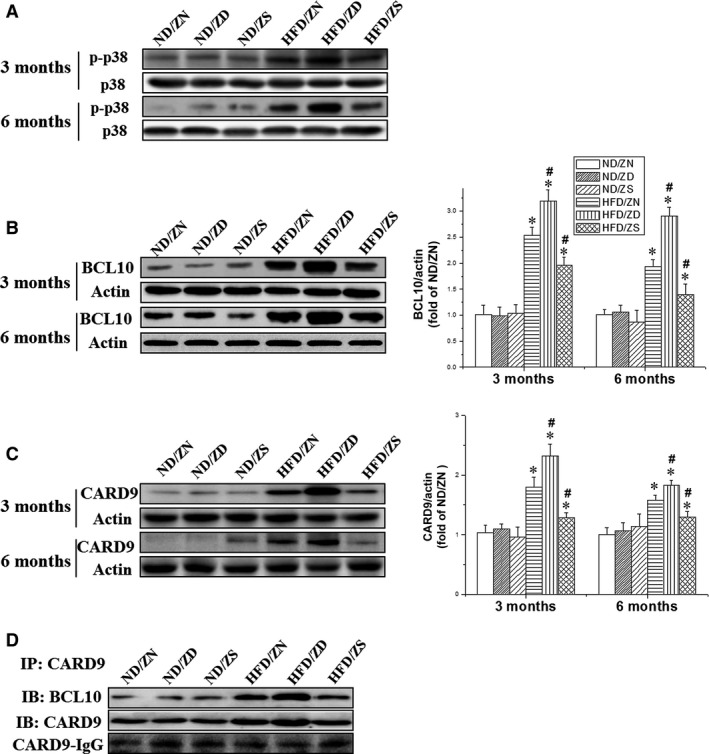
Effect of zinc status on HFD‐induced phosphorylation of p38 MAPK and expression of BCL10/CARD9**.** (**A**) Expression of phosphorylation of p38 MAPK at 3 months and 6 months was examined with Western blots. (**B** & **C**) Expression of BCL10 and CARD9 at 3 months and 6 months was examined with Western blots (left) and quantified (Origin 8.0 software) (right). Actin was used as a control. Data were presented as mean ± S.D. (*n* = 5–8 per group). *P < 0.05 *versus* ND/ZN group; #*P* < 0.05 *versus* HFD/ZN group. (**D**) Co‐immunoprecipitation was performed with 500 μg protein purified from left ventricles of mice from various groups as indicated. IP, anti‐CARD9 antibody; IB, anti‐BCL10 and anti‐CARD9.

In our previous study, we found that HFD‐induced ORCH and p38 MAPK activation were exacerbated and prevented by zinc deficiency and zinc supplement, respectively 6. Inhibition of p38 MAPK prevented ORCH in HFD‐induced obese mice. To explore how obesity activates p38 MAPK, and whether BCL10 and CARD9 expression increases in parallel to p38 MAPK phosphorylation, the levels of expression of these proteins were examined in HFD‐induced obese mice. In parallel to p38 MAPK phosphorylation (Fig. 1A), the expression of BCL10 and CARD9 increased in HFD/ZN mice at both 3 and 6 months (Fig. 1B and C). HFD/obesity‐induced expression of both BCL10 and CARD9 further increased due to zinc deficiency. This effect was significantly reduced in zinc‐supplemented animals (Fig. 1B and C).

Previous studies reported that the BCL10–CARD9 complex was required for the activation of their downstream signalling pathways, such as NF‐κB‐mediated inflammation 7. We next examined the effect of zinc on the formation of BCL10–CARD9 complex by co‐immunoprecipitation (co‐IP). Co‐IP was performed with anti‐CARD9 antibody to collect CARD9 protein complexes. Anti‐BCL10 and anti‐CARD9 antibodies were used to confirm the presence of the BCL10–CARD9 complex. Although the amount of CARD9 was significantly elevated in both the HFD/ZN and HFD/ZD hearts, the amount of BCL10 in the complex was primarily elevated in the HFD/ZD group compared to the HFD/ZN group (Fig. 1D). This finding suggests that zinc may mediate the dissociation of BCL10–CARD9 complex in HFD mice.

### The expressions of cardiac BCL10 and CARD9 were not affected by p38 MAPK inhibition

**Figure 2:**
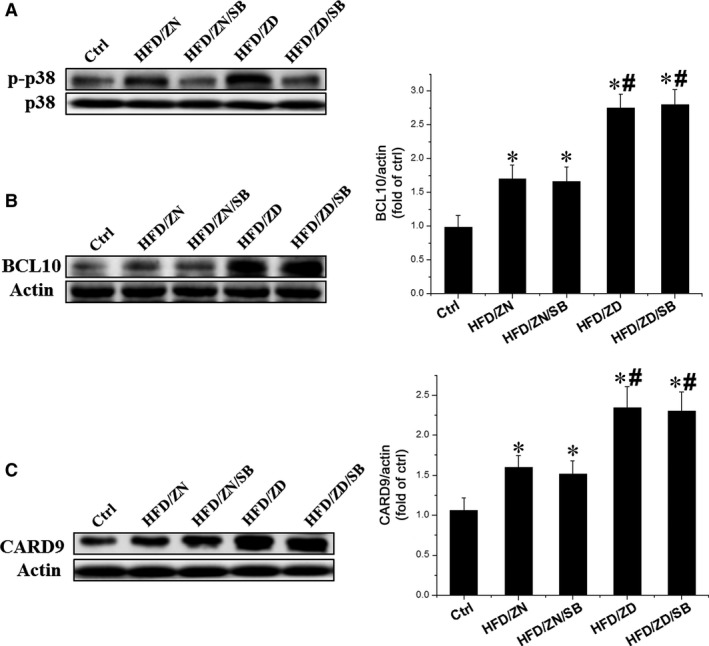
Effect of p38MAPK inhibitor on the expressions of p38 MAPK, BCL10 and CARD9. Western blotting was performed on protein lysates purified from the hearts of mice from various treatment groups as indicated. (**A**) Phosphorylation of p38 MAPK was examined by Western blotting. Total p38 was used as a loading control. (**B** & **C**) Expressions of BCL10 (**B**, left) and CARD9 (**C**, left) were measured by Western blotting. The right panels were the statistical analysis of expression levels of BCL10 (**B**) and CARD9 (**C**). Data were presented as mean ± S.D. (*n* = 6–8 in each group). *P < 0.05 *versus* Ctrl group; #*P* < 0.05 *versus* HFD/ZN group; &, *P* < 0.05 *versus* HFD/ZD group.

Significant inflammation and cardiac hypertrophy were blocked by the inhibition of p38 MAPK in obese mice 22. The levels of p38 MAPK, BCL10 and CARD9 in obese mouse hearts were significantly higher than those from ND/ZN mice (Fig 2A–C). Treatment with SB203580 inhibited p38 MAPK phosphorylation, but did not significantly reduce the expression of BCL10 and CARD9 in the hearts of HFD mice compared to ND/ZN mice (Fig. 2B and C). Collectively, these findings suggest that BCL10 and CARD9 may be upstream of p38 MAPK or function in a parallel pathway.

### Zinc depletion exacerbated palmitate‐induced cardiomyocyte hypertrophy through BCL10‐mediated activation of p38 MAPK

**Figure 3:**
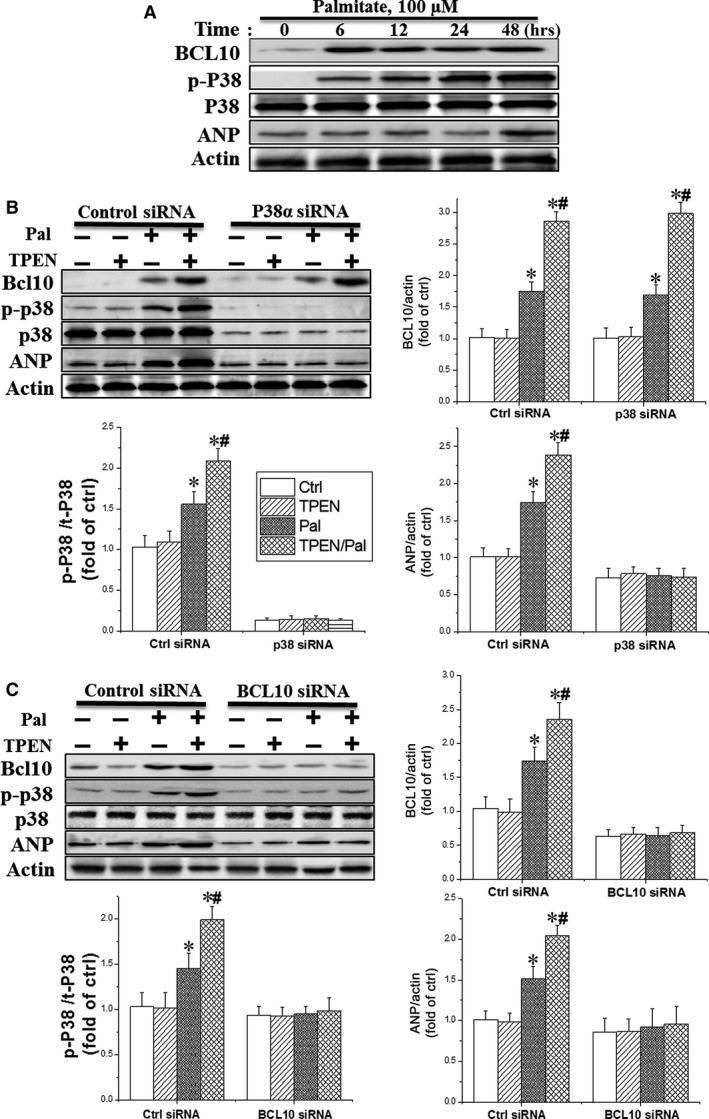
BCL10 was required for palmitate‐induced activation of p38 AMPK in ORCH. (**A**) Cultured mouse cardiomyocytes were pre‐treated with Pal (100 μM) at different time‐points (0, 6, 12, 24 and 48 hrs). (**B** & **C**) Primary cardiomyocytes were pre‐treated with TPEN (2 μM) or 1 × PBS for 1 hr followed by treatment with Pal (100 μM) in the presence of siRNA against p38 MAPK (**B**), BCL10 (**C**) or control siRNA as indicated. P38 MAPK phosphorylation and the expression of BCL10 and ANP were examined by Western blot. The corresponding statistical analysis was shown on the left. Data were presented as mean ± S.D. from at least three separate experiments. *P < 0.05 *versus* Ctrl group; #*P* < 0.05 versus Pal group (Origin 8.0 software).

We next examined the relationship between the BCL10/CARD9 complex formation and p38 MAPK activation *in vitro* using cultured cardiomyocytes. As BCL10/CARD9 complex formation was required for activation of the downstream targets 7, 16, we suppressed BCL10 to reduce the activity of this complex. As shown in Fig. 3A, palmitate at 100 μM increased the expression of BCL10 at 6–12 hrs, whereas p38 MAPK upregulation occurred at 24–48 hrs and ANP at 48 hrs.

To confirm the relationship between BCL10 and p38 MAPK in an obese model, we knocked down p38 MAPK and BCL10 in cardiomyocytes using siRNA. Removal of zinc from the medium, using the zinc chelator TPEN combined with palmitate treatment further increased the phosphorylated p38 MAPK and ANP levels, compared to palmitate treatment alone. This indicates that zinc deficiency up‐regulates p38 MAPK to worsen cardiac hypertrophy. Compared to control siRNA, specific p38 MAPK siRNA effectively knocked down the expression and the phosphorylation of p38 MAPK. However, p38 MAPK siRNA did not affect BCL10 expression, but significantly reduced palmitate‐ or TPEN/palmitate‐induced expression of ANP (Fig. 3B).

The association of p38 MAPK and ANP expressions with of BCL10 was confirmed using specific BCL10 siRNA to knockdown the kinase in adult primary cardiomyocytes (Fig. 3C). Decreased levels of BCL10 suppressed palmitate or TPEN/palmitate‐induced p38 MAPK phosphorylation and ANP expression. As a negative control, the expressions of 3‐nitrotyrosine (3‐NT) and 4‐hydroxy‐2‐nonenal (4‐HNE) (Fig. S1A and B) were not affected by the suppression of BCL10, confirming the specificity of p38 MAPK and ANP by BCL10 siRNA. Taken together, we conclude that BCL10 is an important mediator of p38 MAPK activation to induce cardiac hypertrophy under obese conditions with or without zinc deficiency.

### Zinc supplement up‐regulated metallothionein in obese mice heart

**Figure 4:**
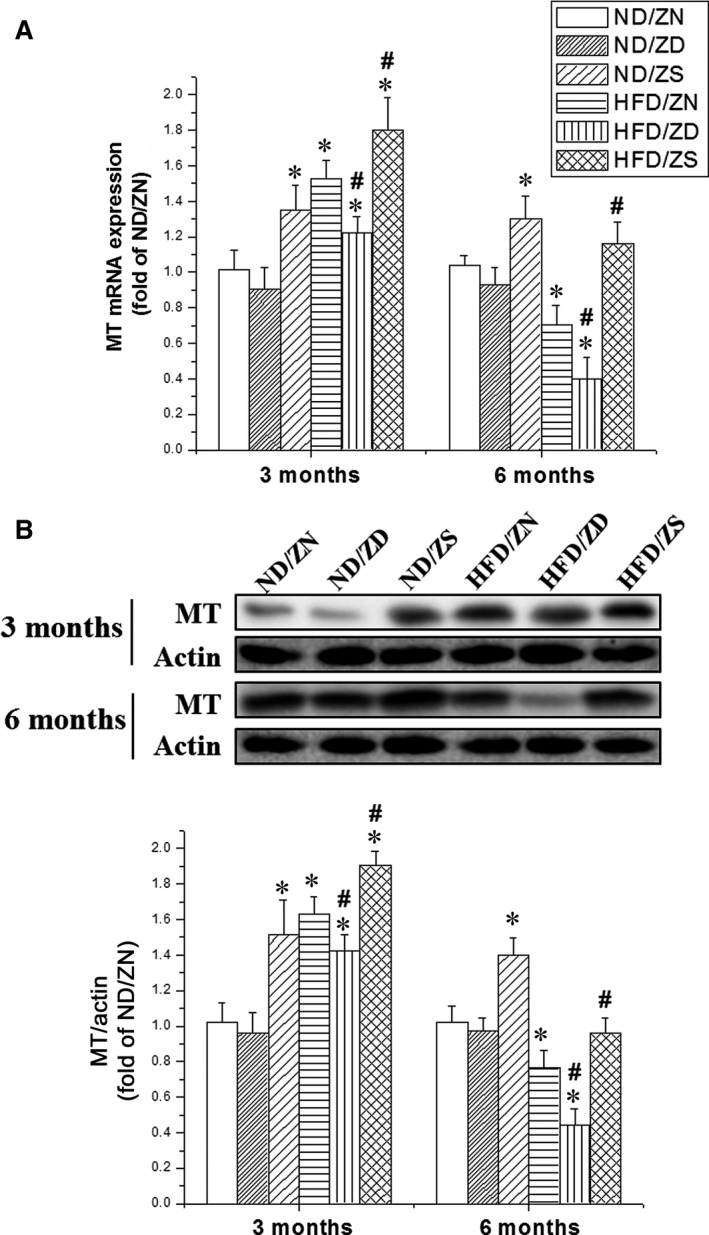
Zinc‐induced MT expression in mouse hearts. The expression of MT was determined by real‐time PCR (**A**) and Western blot (**B**) at both 3 and 6 months, respectively. Actin was used as a control for both mRNA and protein. The lower panel of B shows the statistical analysis of the protein expression of MT in different treatment groups as indicated. Data were presented as mean ± S.D. (*n* = 5–8 in each group). *P < 0.05 *versus* ND/ZN group; #*P* < 0.05 *versus* HFD/ZN group.

**Figure 5:**
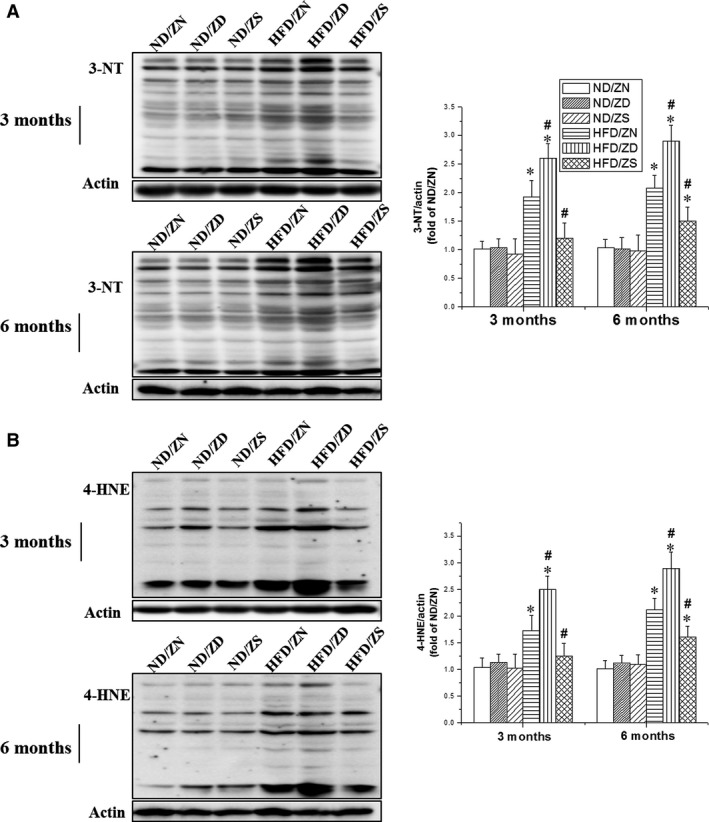
Impacts of zinc status on HFD‐triggered cardiac oxidative damage. Western blotting was performed to measure the expression of 3‐NT (**A**) and 4‐HNE (**B**) to assess the nitrosative and oxidative damage in the heart at 3 months and 6 months, respectively. The corresponding statistical analysis was shown on the left of each panel. Actin was used as a control. Data were presented as mean ± S.D. (*n* = 5–8 in each group). *P < 0.05 *versus* ND/ZN group; #*P* < 0.05 *versus* HFD/ZN group.

It is known that zinc is an inducer of MT 19, which is an important endogenous antioxidant. We observed significant elevations in the expression of MT mRNA in the heart of zinc‐treated control mice at 3 and 6 months (Fig. 4A). HFD‐induced obesity also increased cardiac MT expression at 3 months, but significantly decreased it at 6 months, which was worsened by zinc deficiency and revised by zinc supplement (Fig. 4A). These mRNA profiles were in agreement with the protein levels of MT as measured by Western blot (Fig. 4B). As expected, HFD‐induced obesity significantly increased cardiac oxidative stress, as evaluated by the quantification of nitrosative stress indicator 3‐NT and lipid peroxidation indicator 4‐HNE, at both 3 and 6 months (Fig. 5A and B). Additionally, zinc deficiency significantly enhanced cardiac oxidative stress, which was markedly attenuated by zinc supplementation.

### p38 MAPK inhibition did not affect the obesity and zinc‐induced decrease in cardiac metallothionein expression

**Figure 6:**
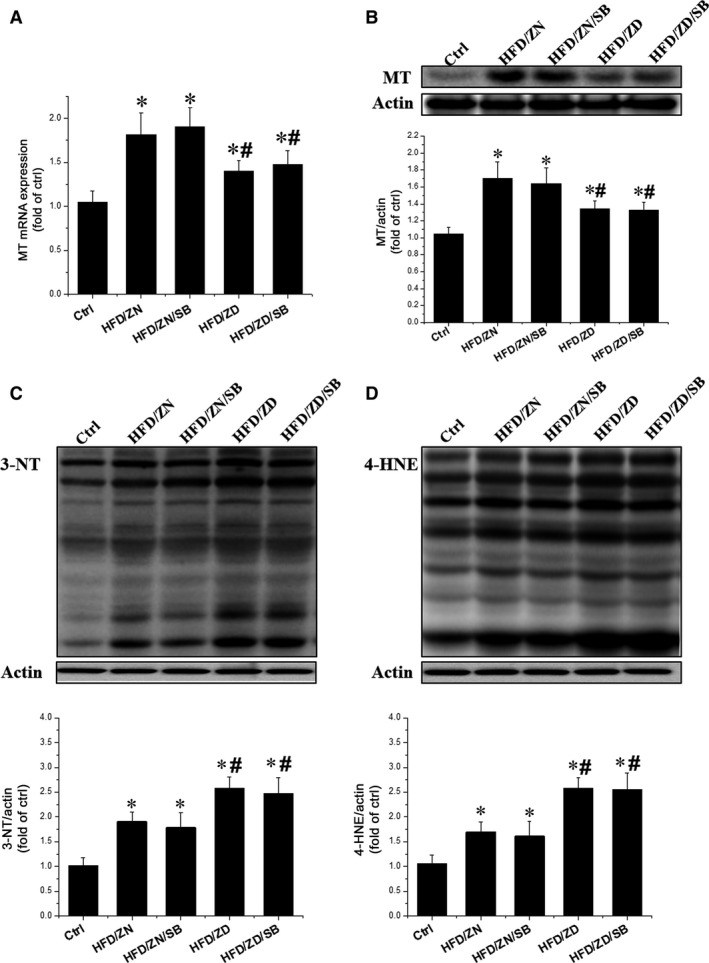
Effect of p38 MAPK inhibitor on MT expression. The mRNA expression (**A**) and protein level (**B**) of MT were detected by PCR and Western blot, respectively. 3‐NT (**C**) and 4‐HNE (**D**) were measured by Western blotting. The lower panels of **C** and **D** were the statistical analysis of 3‐NT and 4‐HNE, respectively. Data were presented as mean ± S.D. (*n* = 6–8 in each group). *P < 0.05 *versus* Ctrl group; #*P* < 0.05 *versus* HFD/ZN group; &*P* < 0.05 *versus* HFD/ZD group.

We investigated whether inhibition of p38 MAPK could prevent decreased MT expression associated with obesity and zinc deficiency. Surprisingly, SB 203580 did not significantly alter the expression of MT at either mRNA or protein levels (Fig. 6A and B). The expression of 3‐NT and 4‐HNE was also not affected by SB 203580 (Fig. 6C and D). Collectively, zinc‐altered expression of MT is independent of the p38 MAPK signalling pathway.

### Zinc inhibition of palmitate‐induced BCL10 expression and p38 MAPK activation was MT‐dependent

**Figure 7:**
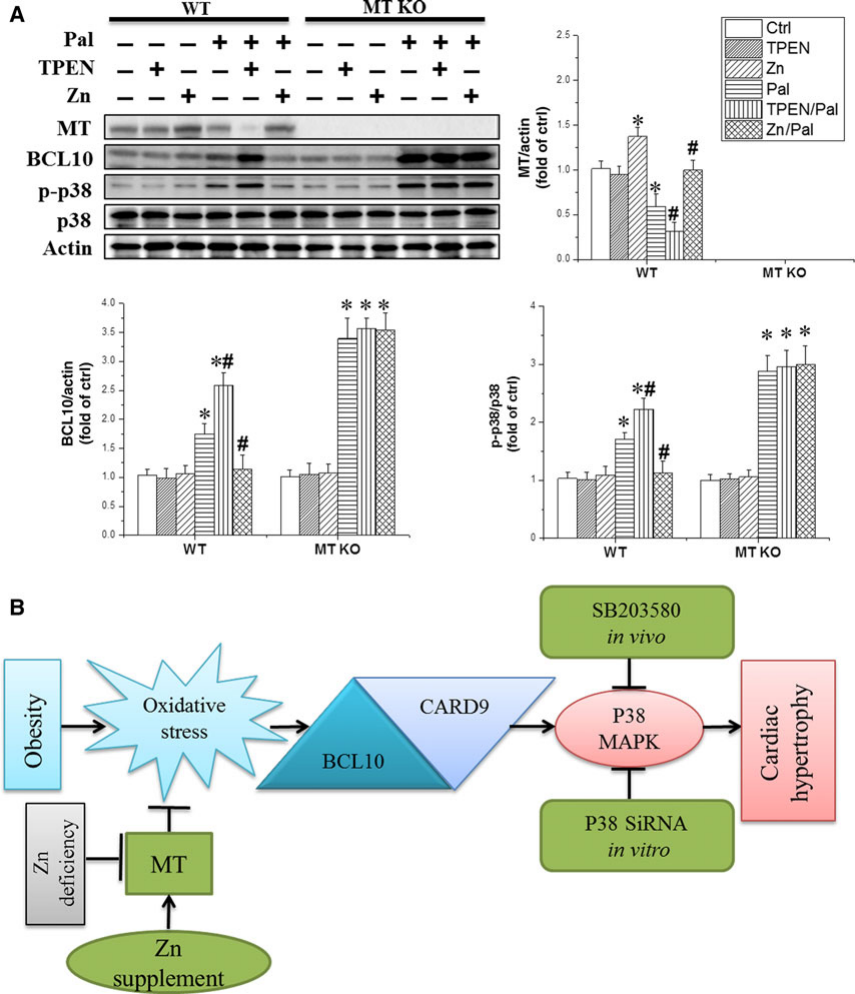
Zinc failed to suppress BCL10 expression and p38 MAPK phosphorylation in the absence of MT **.** (**A**) Primary cultured cardiomyocytes were pre‐treated with TPEN (2 μM) or 1 × PBS for 1 hr followed by co‐treatment with palmitate (100 μM) with or without zinc chloride (50 μM) in MT‐KO cardiomyocytes. The expression of MT, BCL10 and p38 MAPK phosphorylation in MT‐KO cardiomyocytes was examined by Western blot. Data were presented as mean ± S.D. from at least three independent experiments. *P < 0.05 *versus* Ctrl group; #*P* < 0.05 versus Pal group. (**B**) Illustration of the potential mechanism by which zinc ameliorates ORCH. Obesity induces BCL10/CARD9 up‐regulation to activate p38 MAPK, which subsequently contributes to the development of ORCH. Zinc deficiency further enhances the activation of BCL10/CARD9/p38 MAPK, while zinc supplement functions through MT to suppress the expression of BCL10/CARD9/p38 MAPK. Inhibition of p38 MAPK with SB203580 completely abolishes the ORCH but does not affect the expression of BCL10/CARD.

We investigated whether MT was involved in zinc‐mediated BCL10 expression and p38 MAPK activation using cardiomyocytes from MT knockout mice. In WT cardiomyocytes, palmitate increased the levels of phosphorylated p38 MAPK and BCL10, which were further elevated by TPEN plus palmitate treatment. As expected, zinc treatment blocked p38 MAPK activation and increased BCL10 expression. In contrast, these responses were not observed in following zinc supplement in MT‐KO mouse cardiomyocytes (Fig. 7A). Additionally, the expression of 3‐NT and 4‐HNE was not reduced by the zinc supplement in MT‐KO cardiomyocytes (Fig.S2A and B). Thus, we conclude that MT is involved in zinc‐mediated BCL10 expression and p38 MAPK activation.

## Discussion

The results presented in this report indicate that 1) HFD‐induced upregulation of BCL10/CARD9 and p38 MAPK activation are enhanced by zinc deficiency, whereas zinc supplement significantly decreases this response; 2) inhibiting p38 MAPK activity in HFD mice or palmitate‐treated cardiomyocytes block the ability of HFD and zinc deficiency to enhance p38 MAPK activation; 3) inhibition of BCL10 expression prevents palmitate‐induced p38 MAPK activation and hypertrophic response; 4) inhibition of p38 MAPK reduces the hypertrophic response in cardiomyocytes but does not significantly suppress BCL10 expression; and 5) deletion of MT blocked zinc supplement‐inhibited BCL10 expression and p38 MAPK activation.

Up‐regulation of phospho‐p38 MAPK is believed to contribute to the development of cardiac hypertrophy in the hearts of obese mice 30, 31. In a diabetes model, p38 MAPK plays a critical role in diabetes‐induced testicular apoptosis, and zinc deficiency exacerbates the testicular injury 32. In addition, zinc‐deficient neuronal IMR‐32 and promyeloid cells exhibit higher levels of phospho‐p38 MAPK 33, 34. In line with these findings, we found that the levels of phospho‐p38MAPK were significantly elevated in HFD‐induced obese mice in a time‐dependent manner and that zinc deficiency or supplement significantly enhanced or attenuated this response, respectively.

BCL10 plays an important role in the development of B‐cell lymphomas of mucosa‐associated lymphoid tissues. Here, BCL10 binds CARD9 to activate NF‐κB, suggesting that CARD9 is an upstream activator of BCL10 and NF‐kB signalling. Recent work established CARD9 as a central integrator of innate immune cell activation. Various upstream receptors that engage CARD9 signalling are recognized, and CARD9 downstream effector pathways have been identified 35. It is becoming clear that CARD9 is a regulator of BCL10 function. CARD9 could play a role as an upstream signalling molecule that recruits BCL10 through BCL10‐CARD9 interactions 7.

Deletion of BCL10 reduced angiotensin‐II‐induced cardiac remoulding 36, suggesting a role for BCL10 in cardiac remodelling and hypertrophy. A recent report indicated that CARD9 contributed to the remodelling of vein grafts, which was supported by the finding that deletion of CARD9 significantly decreased the NF‐κB phosphorylation induced by exposure of macrophages to necrotic smooth muscle cells 37. Other studies demonstrated that CARD9 signals allowed Toll‐like receptor pathways to regulate p38 MAPK activation, CARD9‐deficient macrophages from Card9 KO‐mice had defects in p38 MAPK activation following bacterial or viral infection 8, 38, 39.

To date, the mechanism by which BCL10/CARD9 activates p38 MAPK remains unclear. p38 MAPK is involved in a number of pathological conditions and is believed to be regulated by the CARD9 signalling complex during innate and adaptive immune responses 8, 40, 41. As one of the downstream transcriptional factors of CARD9 signalling, phosphorylation levels of p38 MAPK significantly increased in isolated macrophages and heart tissue. This increase in p38 MAPK activity may be responsible for the up‐regulation of a number of proinflammatory cytokines in the heart. CARD9 knockout attenuates cardiac dysfunction *via* abrogating the increase in p38 MAPK phosphorylation in obese mice and angiotensin‐II‐induced cardiac inflammation and fibrosis 31, 42. In the present study, zinc deficiency exacerbated BCL10 and CARD9 expression in obese mice, while zinc supplement reduced the BCL10 and CARD9 levels. In addition, the levels of BCL10 and CARD9 increased in obese mice, compared to controls (ND/ZN), and the BCL10/CARD9 complex was significantly increased in obese mouse hearts compared to the control hearts. Interestingly, BCL10 and CARD9 were not affected by the treatment of p38 MAPK inhibitor. In adult primary cardiomyocytes, p38 MAPK knockdown with siRNA completely abolished phospho‐p38 MAPK and ANP expression, but did not affect BCL10 activation, suggesting that BCL10 was upstream of p38 MAPK. The mechanism is further supported by the observation that inhibition of BCL10 suppressed the upregulation of phospho‐p38 MAPK and ANP expression induced by palmitate plus TPEN treatment. Together these results confirm that p38 MAPK functions downstream of BCL10 in ORCH. Furthermore, they support a model where BCL10 and CARD9 are upstream of p38 MAPK, and that their interaction directly activates p38 MAPK to promote cardiac hypertrophy.

Previous studies from our group and others show that zinc supplementation offers benefits to diabetic or non‐diabetic obese patients and in animal models by enhancing insulin sensitivity, anti‐oxidative stress and anti‐inflammation by increasing MT expression 19, 20. Increases in p38 MAPK activity play important role in cardiac apoptotic cell death both *in vivo* and *in vitro*. Furthermore, suppression of MT expression of cardiac apoptotic effect is associated with the inhibition of p38 MAPK activation 43. MT also suppresses the expression of p38 MAPK in arsenic trioxide‐induced cardiotoxicity 44. Therefore, developing pharmaceutical agents that selectively elevate cardiac MT levels to inhibit p38 MAPK activation could be a beneficial strategy for protection against cardiotoxicity. In the present study, zinc up‐regulated MT expression, while p38 MAPK inhibition did not affect expression. Additionally, our *in vitro* study showed that deletion of MT expression abolished the prevention by zinc of BCL10 upregulation and p38 MAPK activation in palmitate‐treated cardiomyocytes. These findings demonstrate that zinc‐attenuated up‐regulation of BCL10 and phosphorylation of p38 MAPK is MT‐dependent. We propose that the zinc supplement‐induced expression of MT may contribute to the diminished expression of BCL10 and phospho‐p38 MAPK (Fig. 7B).

Metallothionein protects the heart against metabolic disorder, through its reduction of oxidative stress 23, 25. Furthermore, ROS can activate Toll‐like receptor under high glucose treatment and in cardiac tissues of diabetic rats *in vivo* 8, 45. Activation of Toll‐like receptor is essential to up‐regulate BCL10 expression and/or CARD9 expression, which in turn activates the p38 MAPK pathway 8, 46, 47, 48. In the present study, we showed that suppression of BCL10 did not affect the reduction in oxidative stress by zinc treatment, suggesting that oxidative stress is upstream of BCL10. Deletion of MT expression blocked the prevention of palmitate‐induced oxidative stress, BCL10/CARD9 expression and p38 MAPK activation by zinc supplement. Therefore, our proposed mechanism is that zinc supplement induces MT expression, to prevent obesity‐induced oxidative stress and the associated consequences (BCL10/CARD9 expression, p38 MAPK activation and hypertrophic signalling), as illustrated in Fig. 7B.

There are significant worldwide increase in the number of obese children and adolescents. Cardiac abnormalities, including cardiac hypertrophy and dysfunction, have been reported in the obese. However, there were only few studies on the cardiovascular effect for the obese children and adolescents 49. Therefore, it is an urgent need to mechanistically understand the pathogenic effect of the obesity in these children and adolescents on their heart. It is the present study to model the cardiac abnormalities of childhood obesity with HFD‐induced obese mice at a young age. Our findings of the significant induction of cardiac hypertrophy and inflammatory responses in the obese mice are consistent with these two studies 49, 50. We further demonstrated that these obesity‐related cardiac pathogenic effects were further exacerbated by zinc deficiency and alleviated by zinc supplement, respectively.

In conclusion, we demonstrate that zinc plays a critical role in modulating the expression of BCL10 and CARD9 associated by HFD, which stimulated p38 MAPK phosphorylation and p38 MAPK‐mediated cardiac hypertrophy signalling. Our *in vitro* results showed that preventing up‐regulation of BCL10 expression and p38 MAPK phosphorylation by zinc supplement is MT‐dependent. Therefore, our findings support the hypothesis that activation of BCL10/CARD9/p38 MAPK‐mediated cardiac hypertrophy pathways plays an important role in the development of ORCH. Furthermore, zinc supplement can attenuate this response and cardiac hypertrophy signalling.

## Conflict of interest

The authors declare no conflict of interest.

## Author contributions

SW completed the research. JG, ZX, ZZ, TB, JC, Q‐JL and JX were involved in part of the experiments. GB, JHF, QL, YZ and LC contributed to the initial discussion and design of the project. SW, JG, ZX and LC drafted the manuscript. JC, GB, JHF, QL, YZ and LC discussed and edited the manuscript. YZ, ZZ, Q‐JL and LC provided funding and reviewed the manuscript.

## Supporting information


**Data S1** Materials and methods.
**Figure S1** 3‐NT and 4‐HNE expression were measured by western blotting.
**Figure S2** Primary cardiomyocytes were pre‐treated with TPEN (2 µM) or 1 × PBS for 1 hr followed by co‐treatment with palmitate (100 µM) with or without Zn Chloride (50 µM) in MT‐KO mice.
