# Supplementary material for: Zinc rescues obesity‐induced cardiac hypertrophy via stimulating metallothionein to suppress oxidative stress‐activated BCL10/CARD9/p38 MAPK pathway
Source: J Cell Mol Med. 2017 Feb 3;21(6):1182–92. doi: 10.1111/jcmm.13050 (PMC5431126; doi:10.1111/jcmm.13050)
Supplement: Supplementary file 1 — Data S1 Materials and methods. Figure S1 3‐NT and 4‐HNE expression were measured by western blotting. Figure S2 Primary cardiomyocytes were pre‐treated with TPEN (2 µM) or 1 × PBS for 1 hr followed by co‐treatment with palmitate (100 µM) with or without Zn Chloride (50 µM) in MT‐KO mice. [file JCMM-21-1182-s001.doc]

**Supporting Information**

**1. Materials and methods**

1.1. Western blotting

Regular Western blots were performed as described previously . Heart tissues were homogenized in RIPA lysis buffer (Santa Cruz Biotechnology, Santa Cruz, CA), and proteins were collected by centrifuging at 12,000 rpm at 4°C for 15 min in a BeckmanGS-6R centrifuge. The protein concentration was measured by Bradford assay (BIO-RAD, Hercules, CA). The protein samples, diluted in loading buffer and heated at 95°C for about 5 min, were then subjected to electrophoresis on 10% sodium dodecyl sulfate polyacrylamide gel electrophoresis (SDS-PAGE) at 110V, followed by transfer onto a nitrocellulose membrane. The membrane was then blocked in blocking buffer (5% milk and 0.5% BSA) for 1 h, followed by incubation with the primary antibodies at different dilution of 1:1,000 – 1:3,000 at 4°C overnight. The primary antibodies included anti-B cell CLL/lymphoma 10 (BCL10), anti-caspase recruitment domain–containing 9 (CARD9), anti-phospho-p38MAPK (Thr 180/Tyr 182), and anti-p38MAPK, all of which were purchased from Cell Signaling (Danvers, MA, USA). Antibodies against β-actin and atrial natriuretic peptide (ANP) were purchased from Santa Cruz Biotech (Santa Cruz, CA, USA). 3-nitrotyrosine (3-NT, Millipore, Billerica, MA), 4-hydroxy-2-nonenal (4-HNE, Alpha Diagnostic International, San Antonio, TX), After unbound antibodies were washed out with Tris-buffered saline (pH 7.2) containing 0.05% Tween-20, membranes were incubated with each corresponding secondary antibody for 1 h at room temperature. The metallothionein (MT) expression was detected using a modified Western Blot protocol, as previously described , with an antibody against MT (Dako, Carpinteria, CA, 1:1500 dilution). The protein bands were analyzed using the BIO-RAD ChemiDoc^TM^ Touch Imaging System (BIO-RAD, Hercules, CA).

1.2. Quantitative real-time PCR

RT-PCR was performed and the mRNA levels of MT in the mouse hearts was quantified by quantitative real-time PCR (qPCR) . Briefly, total RNA was extracted from testicular tissues using TRIzol reagent (RNA STAT 60 Tel-Test Ambion, Austin, TX). The cDNA was synthesized from 1 μg of total RNA according to the manufacturer's protocol of RNA RCR kit (GoTaq^R^ qPCR Master Mix, LOT0000094718, Promega Corporation, Madison, WI). The primers (MT Mm00496660_g1, β-actin: Mm00607939) were from Applied Biosystems (Carlsbad, CA). The expression levels of target genes were normalized to that of the housekeeping gene β-actin.

1.3. Co-immunoprecipitation of BCL10-CARD9 complex

Heart tissue (40 mg) from each mouse was lysed in immunoprecipitation buffer (25mM Tris, pH 7.6; 150 mM NaCl; 1 mM EDTA; 1% NP-40; protease and phosphatase inhibitors). Pre-washed supernatants (300 μg) were immunoprecipitated overnight at 4 °C with the specific monoclonal antibodies CARD9 (Cell signaling, Danvers, MA, USA) pre-absorbed to protein G-agarose (Pierce Biotechnology Ltd., Rockford, IL, USA). The resulting immunoprecipitates were extensively washed with lysis buffer, eluted with SDS loading buffer by boiling for 5 min, and detected by Western blot with the indicated antibodies. Data were collected from at least three independent experiments.

1.4. Isolation of adult mouse cardiomyocytes, cell culture, palmitate and TPEN treatments, and siRNA transfection

Two-month old mice were anesthetized with ketamine (43.5 mg/kg), acepromazine (1.5 mg/kg) and xylazine (1.7 mg/kg), and given heparin (100U/mL, i.p.). After median sternotomy, the heart was rapidly excised and rinsed with physiological saline. The aortic lumen was tied to an 18-g cannula and perfused with tyrode bicarbonate buffer at 37°C for 5 min. The perfusate was then changed to 50 mL of recirculating LiberaseBlendzyme type 1 (Roche) digestion buffer for 12–15 min. The heart tissue was removed, shredded and filtered through a 140 µm nylon mesh. The supernatant was transferred to another tube and then CaCl_2_ was added in a graded fashion at 4-min intervals (five total steps) to sequentially increase the Ca^2+^ concentration to 500 µM. The suspension was then plated on laminin-coated culture dishes overnight at 37°C in a 5% CO2 incubator. The media was replaced before experimentation to wash away unattached cells and ensure that only rod-shaped myocytes were used for subsequent studies.

Palmitate (Pal, Sigma Aldrich, MO) was dissolved in 50% ethanol, heated at 70°C for 2 min, and added to 2% fatty acid–free BSA (Sigma-Aldrich) in medium as stock solution (2.5 mM). Before use, the stock Pal solution was gently rotated for 1 h at 37°C and further diluted in 1 x phosphate buffered saline (PBS) to the required concentrations for treatment. The recombinant TPEN was dissolved in 1 x PBS to the required concentration. In the concentration-dependent study, adult mouse cardiomyocytes were pre-treated with different doses of Pal for 48 h. In the time course study, mouse cardiomyocytes were pre-treated with Pal (100µM) for different times (0, 6, 12, 24, 48 h). The siRNA transfections in adult mouse cardiomyocytes were performed using Lipofectamine^TM^ 2000 (Invitrogen, Carlsbad, CA) following the manufacturer’s instructions. Briefly, 1) 8.0 μg siRNA was diluted in 0.5 ml of MEM Medium without serum and mixed gently; 2) Lipofectamine™ 2000 was mixed gently before use, and then 20 μL lipofectamine 2000 was diluted in 0.5 mL of MEM medium and incubated for 5 min at room temperature; 3) the diluted siRNA was combined with diluted Lipofectamine™ 2000 (total volume = 1 mL), which was mixed gently and incubated for 20 min at room temperature; 4) 1 mL of complexes was then added to each well containing cells and medium, which was mixed gently by rocking the plate back and forth; 5) after incubation cells at 37°C in a CO_2_ incubator for 4 h, the transfection medium was replaced by MEM culture medium with 10% serum, and the cells were allowed to grow for 32 h prior to testing for transgene expression.

In order to knock down p38 MAPK and BCL10 in adult mouse cardiomyocytes, the primary cultured cardiomyocytes were transfected with mouse non-targeting Stealth RNAi™ siRNA (120 nM) along with the corresponding negative control siRNAs (120 nM) (Invitrogen, Carlsbad, CA). After transfection of siRNA, the primary cardiomyocytes were treated with N,N,N′,N′, tetrakis (2-pyridylmethyl) ethylenediaminepentaethylene (TPEN) (2 μM) or Zn Chloride (50 μM) for 1 h, followed by co-treatment with Pal (100 μM) for another 48 h. The knockdown efficiency was assessed by Western blot based on the target protein expression.

**2.SupplementaryFigures**

**
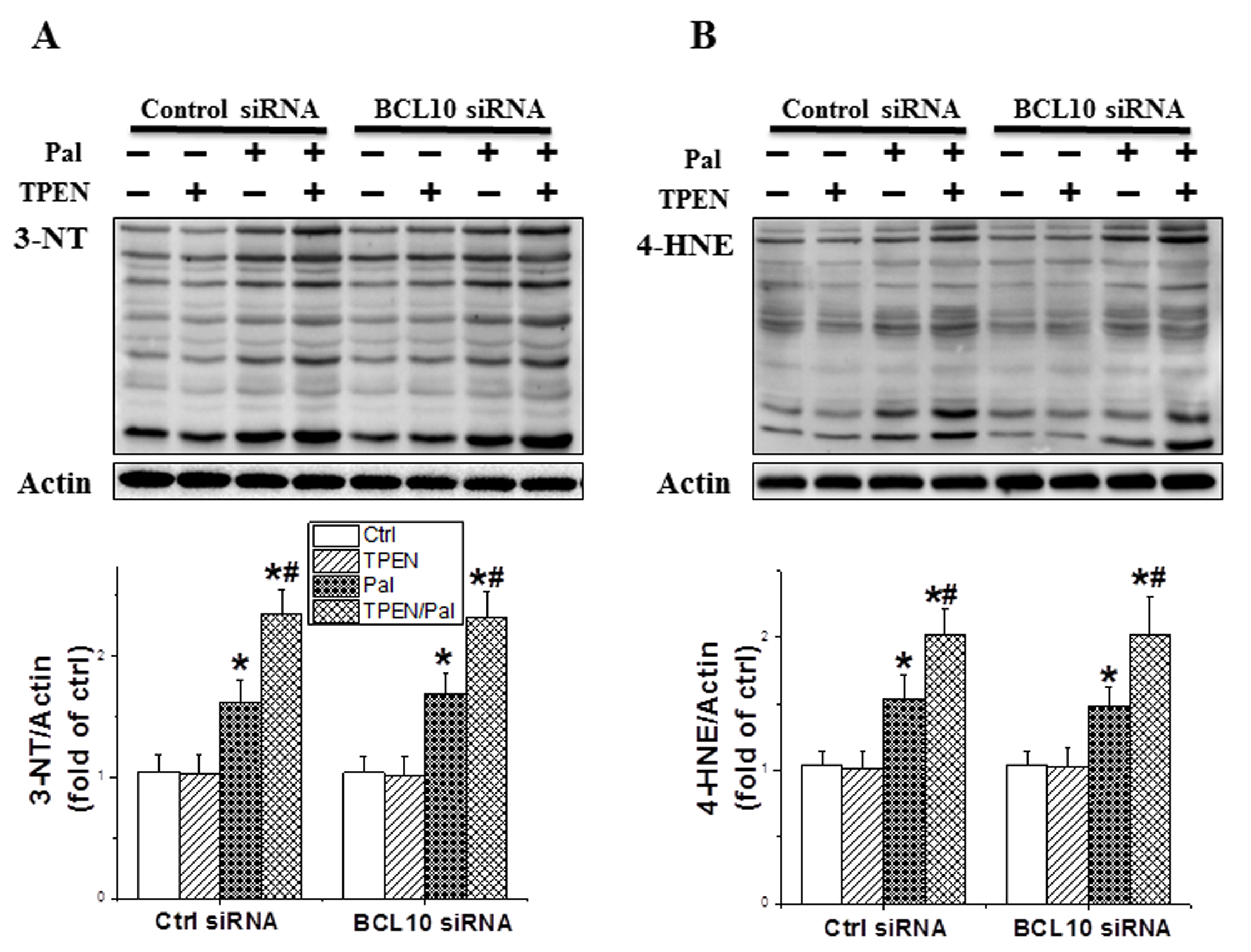
**

**Figure S1.** 3-NT and 4-HNE expression were measured by western blotting. Primary cardiomyocytes were pre-treated with TPEN (2 µM) or 1 x PBS for 1 h followed by co-treatment with pal (100 µM) in the presence of siRNA against BCL10 or control siRNA. 3-NT and 4-HNE were examined by western blot. Data were presented as means ± SD from at least three separate experiments. *, p<0.05 vs. Ctrl group; #, p<0.05 vs. TPEN group.


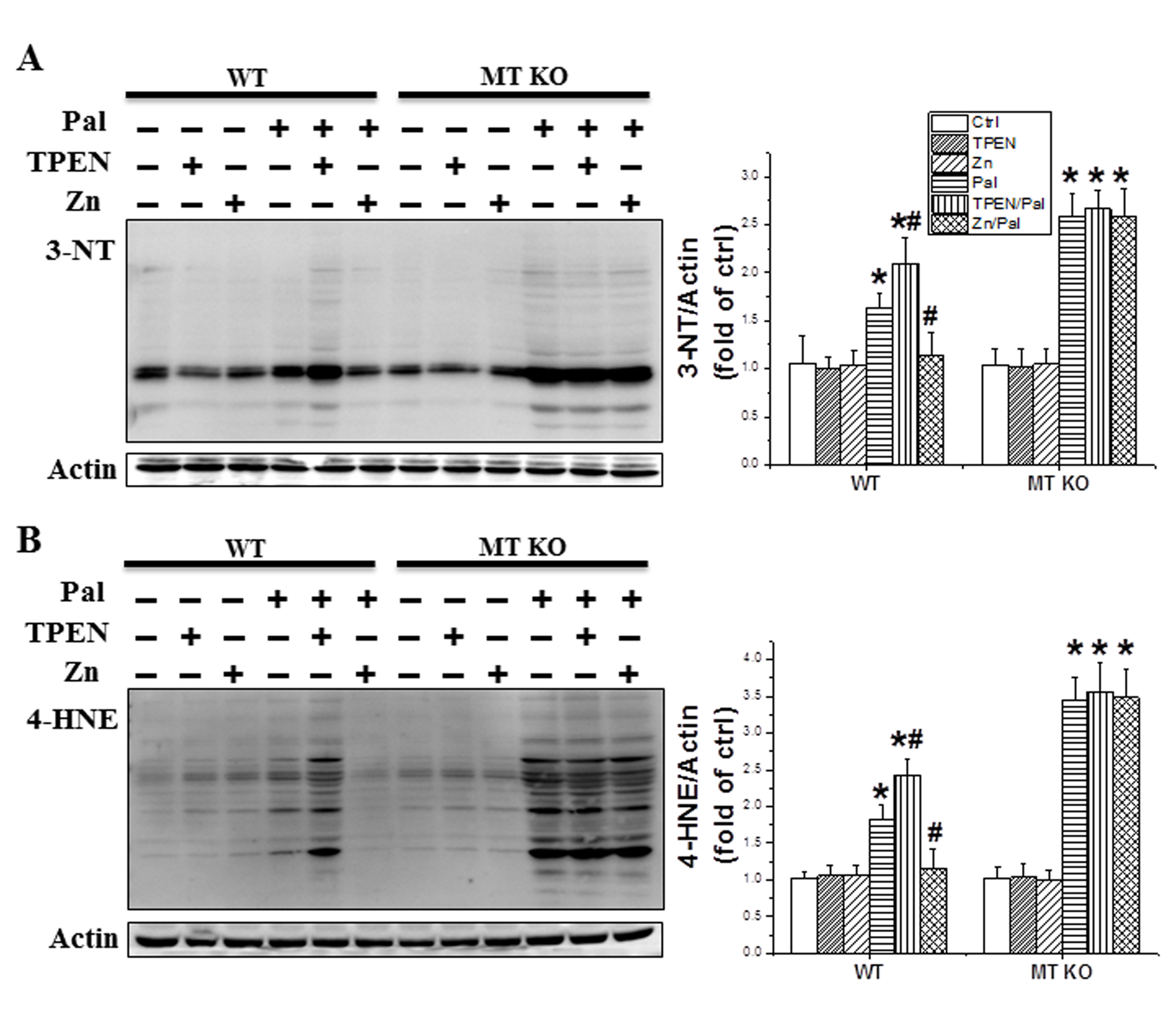


**Figure S2.** Primary cardiomyocytes were pre-treated with TPEN (2 µM) or 1 x PBS for 1 h followed by co-treatment with palmitate (100 µM) with or without Zn Chloride (50 µM ) in MT-KO mice. The expression of 3-NT and 4-HNE in MT-KO mouse hearts were examined by Western blot. Data were presented as mean ± SD from at least three separate experiments. *, p<0.05 vs. Ctrl group; #, p<0.05 vs. TPEN group.

**References**

1. **Zhang Z, Wang S, Zhou S, et al.** Sulforaphane prevents the development of cardiomyopathy in type 2 diabetic mice probably by reversing oxidative stress-induced inhibition of LKB1/AMPK pathway. *J Mol Cell Cardiol*. 2014; 77: 42-52.

2. **Wang J, Song Y, Elsherif L, et al.** Cardiac metallothionein induction plays the major role in the prevention of diabetic cardiomyopathy by zinc supplementation. *Circulation*. 2006; 113: 544-54.

3. **Zuo YF, Gu J, Qiao Z, et al.** Effects of dry method esterification of starch on the degradation characteristics of starch/polylactic acid composites. *Int J Biol Macromol*. 2015; 72: 391-402.
